# All-in-one adeno-associated virus delivery and genome editing by *Neisseria meningitidis* Cas9 in vivo

**DOI:** 10.1186/s13059-018-1515-0

**Published:** 2018-09-19

**Authors:** Raed Ibraheim, Chun-Qing Song, Aamir Mir, Nadia Amrani, Wen Xue, Erik J. Sontheimer

**Affiliations:** 1 RNA Therapeutics Institute, University of Massachusetts Medical School, Worcester, MA 01605 USA; 2 Program in Molecular Medicine, University of Massachusetts Medical School, Worcester, MA 01605 USA

**Keywords:** CRISPR, All-in-one rAAV, Genome editing, NmeCas9

## Abstract

**Background:**

Clustered, regularly interspaced, short palindromic repeats (CRISPR) and CRISPR-associated proteins (Cas) have recently opened a new avenue for gene therapy. Cas9 nuclease guided by a single-guide RNA (sgRNA) has been extensively used for genome editing. Currently, three Cas9 orthologs have been adapted for in vivo genome engineering applications: *Streptococcus pyogenes* Cas9 (SpyCas9), *Staphylococcus aureus* Cas9 (SauCas9), and *Campylobacter jejuni* (CjeCas9). However, additional in vivo editing platforms are needed, in part to enable a greater range of sequences to be accessed via viral vectors, especially those in which Cas9 and sgRNA are combined into a single vector genome.

**Results:**

Here, we present in vivo editing using *Neisseria meningitidis* Cas9 (NmeCas9). NmeCas9 is compact, edits with high accuracy, and possesses a distinct protospacer adjacent motif (PAM), making it an excellent candidate for safe gene therapy applications. We find that NmeCas9 can be used to target the *Pcsk9* and *Hpd* genes in mice. Using tail-vein hydrodynamic-based delivery of NmeCas9 plasmid to target the *Hpd* gene, we successfully reprogram the tyrosine degradation pathway in Hereditary Tyrosinemia Type I mice. More importantly, we deliver NmeCas9 with its sgRNA in a single recombinant adeno-associated vector (rAAV) to target *Pcsk9*, resulting in lower cholesterol levels in mice. This all-in-one vector yielded > 35% gene modification after two weeks of vector administration, with minimal off-target cleavage in vivo.

**Conclusions:**

Our findings indicate that NmeCas9 can enable the editing of disease-causing loci in vivo, expanding the targeting scope of RNA-guided nucleases.

**Electronic supplementary material:**

The online version of this article (10.1186/s13059-018-1515-0) contains supplementary material, which is available to authorized users.

## Background

A major advance in the field of gene therapy has been the introduction of Cas9 nuclease-enabled genome editing [1]. Clustered, regularly interspaced, short palindromic repeats (CRISPR) loci specify an adaptive immune pathway that evolved in bacteria and archaea to defend against mobile genetic elements (MGEs) [2, 3]. The effector complex in type II CRISPR systems includes the Cas9 nuclease, which is guided by a CRISPR RNA (crRNA) and a trans-activating RNA (tracrRNA). These dual RNAs can be fused to form a single-guide RNA (sgRNA) [4]. Each crRNA contains a unique “spacer” sequence that can be programmed to cleave a DNA segment of interest. Cas9 scans DNA for a specific protospacer adjacent motif (PAM), opens the duplex to form an RNA-DNA hybrid between the guide and the spacer, and introduces a double-strand break (DSB) in the DNA target [1, 3]. Cas9 and sgRNA have been adapted to enable genome editing in cultured cells following various modes of delivery including plasmid and RNA transfections, viral transduction, and ribonucleoprotein (RNP) electroporation. Precise and efficient in vivo editing is more difficult to achieve, largely due to the difficulties inherent in delivery.

Several methods have been developed to deliver Cas9 in vivo including viral and non-viral methods [5]. These include the use of gold and lipid nanoparticles to deliver Cas9 in RNP or RNA form in mice. However, these methods present challenges for routine use including cost and tissue distribution [6–8]. One of the more intriguing gene delivery vehicles that has emerged in recent years is recombinant adeno-associated virus (rAAV). This vector possesses several attributes that benefit gene therapy applications, including lack of pathogenicity and replication as well as an ability to infect dividing and non-dividing cells [9]. In addition, rAAV is also capable of infecting a wide range of cells and maintain sustained expression [10, 11]. Compared to other viral vectors, rAAV persists in concatemeric, episomal forms, while eliciting mild immune responses [12–14]. The usefulness of rAAV-based delivery for gene therapy is reflected in the number of clinical trials involving rAAV [15]. One of the most exciting advancements for rAAV gene therapy field has been the FDA’s recent market approval of a therapy for RPE65-mediated inherited retinal disease (IRD), the first of its kind in the United States [16].

More recently, several groups have focused their efforts on using this tool for in vivo delivery of Cas9 orthologs [17–20]. The majority of Cas9 genome editing efforts have been focused on the widely used type II-A ortholog from *Streptococcus pyogenes*, SpyCas9. Although it exhibits consistently robust genome-editing activity, considerable effort has been required to overcome off-target editing activities of wild-type SpyCas9 [21–23] [Amrani et al., manuscript in revision (https://www.biorxiv.org/content/early/2018/05/09/172650)]. Furthermore, its large size (1368 amino acids, 4.10 kb) restricts its delivery with its guide in a single virion with potent vectors such as rAAV [24]. Split SpyCas9 constructs (expressed from separate viruses) have been employed [19], though activity is sometimes compromised [25–27]. Dual-rAAV delivery of SpyCas9 and sgRNA can be achieved [28], but it requires the usage of highly minimized promoters that limit expression and tissue specificity. Furthermore, dual rAAV formats carry significant costs as well as limitations in co-transduction.

Alternatively, compact Cas9 orthologs can be packaged in all-in-one rAAV vectors. Type II-A *Staphylococcus aureus* (SauCas9) (1053 amino acids, 3.16 kb) and type II-C *Campylobacter jejuni* Cas9 (CjeCas9) (984 amino acids, 2.95 kb) have been deployed successfully via rAAV in mice [18, 20]. However, unlike the highly abundant NGG SpyCas9 PAM, these Cas9 nucleases have more restrictive PAM requirements (for SauCas9, 5’-NNGRRT-3′; for CjeCas9, 5’-N_4_RYAC [29]. Furthermore, off-target editing by SauCas9 is not unusual [18, 30]. For these reasons, many genomic sites of interest cannot be targeted by all-in-one rAAV delivery of the Cas9 genome editing machinery and additional capabilities and PAM specificities are therefore needed.

We and others have reported genome editing in mammalian cells by the type II-C Cas9 from *Neisseria meningitidis* strain 8013 (NmeCas9) [31–33] (Amrani et al., manuscript in revision). NmeCas9 is small (1082 amino acids, 3.16 kb), targets PAMs (N_4_GAYT > N_4_GYTT/N_4_GAYA/N_4_GTCT) that are distinct from those of the other compact Cas9 orthologs described above, and is intrinsically resistant to off-targeting [32] (Amrani et al., manuscript in revision). Additionally, NmeCas9 can be subjected to off-switch control by anti-CRISPR proteins [34], which could facilitate spatial and temporal control over NmeCas9 activity in vivo and ex vivo.

In this study, we report the in vivo delivery of NmeCas9 and its guide by a single expression cassette that is sufficiently small for all-in-one rAAV vectors. Two disease genes were targeted separately to highlight the therapeutic potential of NmeCas9: the *Hpd* gene in a hereditary tyrosinemia type I (HTI) mouse model (*Fah*^*neo*^); and the *Pcsk9* gene in C57Bl/6 mice. *Hpd* encodes the 4-hydroxyphenylpyruvate dioxygenase enzyme in the tyrosine metabolism pathway and disrupting *Hpd* can lead to a decrease in the accumulation of toxic fumarylacetoacetate in tyrosinemia models [35]. Separately, *Pcsk9* encodes proprotein convertase subtilisin/kexin type 9 (PCSK9), an antagonist of the low-density lipoprotein (LDL) receptor [36, 37]. When PCSK9 is knocked out, more LDL receptors are available at the surface of hepatocytes to allow cholesterol binding and recycling towards the lysosomes for degradation [38, 39]. The alleviation of tyrosinemia symptoms upon *Hpd* disruption, as well as the reduced serum cholesterol levels that result from *Pcsk9* disruption, provide convenient readouts and benchmarks for genome editing activity [18, 35]. We used these systems to validate all-in-one rAAV delivery of NmeCas9 as an effective in vivo genome editing platform in mammals.

## Results

### Efficient genome editing using all-in-one AAV-sgRNA-hNmeCas9 plasmid in cells and in vivo by hydrodynamic injection

**Fig. 1:**
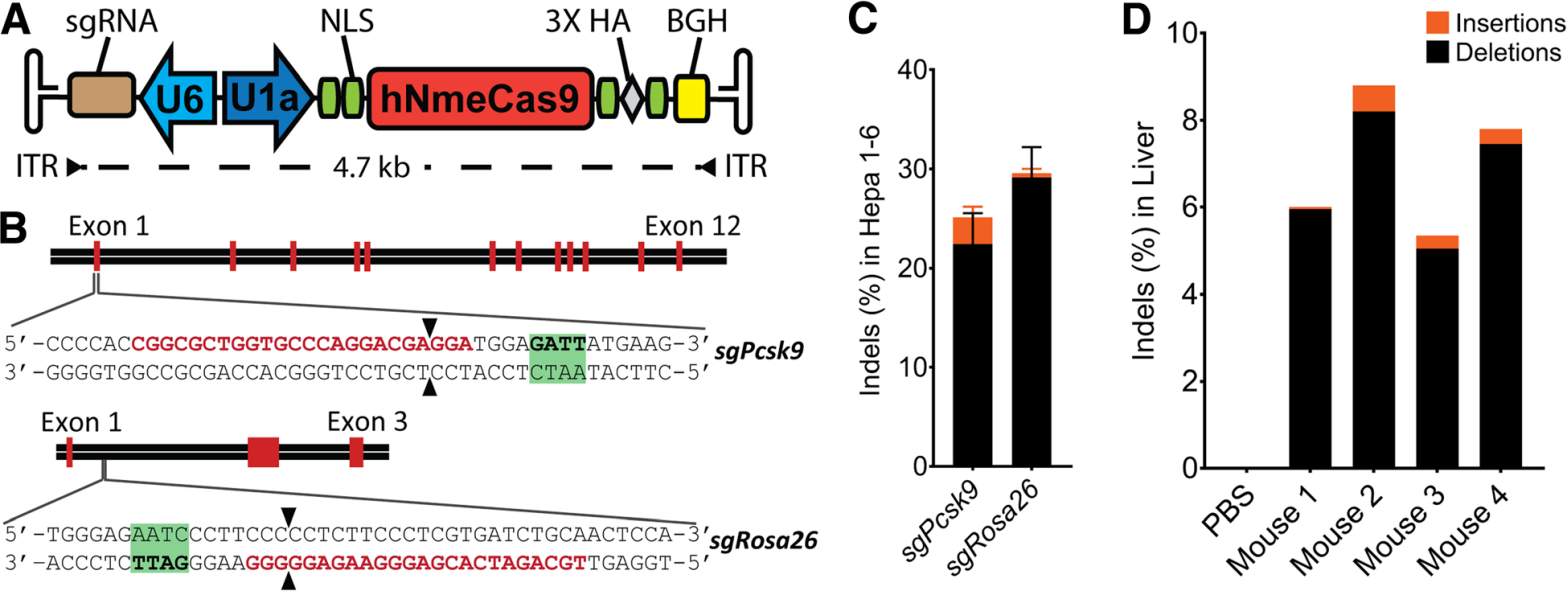
Validation of an all-in-one AAV-sgRNA-hNmeCas9 construct. **a** *Schematic representation* of a single rAAV vector expressing human-codon-optimized NmeCas9 and its sgRNA. The backbone is flanked by AAV inverted terminal repeats (ITR). The poly(a) signal is from rabbit beta-globin (BGH). **b** *Schematic diagram* of the *Pcsk9* (*top*) and *Rosa26* (*bottom*) mouse genes. *Red bars* represent exons. Zoomed-in views show the protospacer sequence (*red*) whereas the NmeCas9 PAM sequence is highlighted in *green*. Double-stranded break location sites are denoted (*black arrowheads*). **c** *Stacked histogram* showing the percentage distribution of insertions-deletions (indels) obtained by TIDE after AAV-sgRNA-hNmeCas9 plasmid transfections in Hepa1–6 cells targeting *Pcsk9* (*sgPcsk9*) and *Rosa26* (*sgRosa26*) genes. Data are presented as mean values ± SD from three biological replicates. **d** *Stacked histogram* showing the percentage distribution of indels at *Pcsk9* in the liver of C57Bl/6 mice obtained by TIDE after hydrodynamic injection of AAV-sgRNA-hNmeCas9 plasmid

Recently, we have shown that the relatively compact NmeCas9 is active in genome editing in a range of cell types (Amrani et al., manuscript in revision). To exploit the small size of this Cas9 ortholog, we generated an all-in-one AAV construct with human-codon-optimized NmeCas9 under the expression of the mouse U1a promoter and with its sgRNA driven by the U6 promoter (Fig. 1a).

Two sites in the mouse genome were selected initially to test the nuclease activity of NmeCas9 in vivo: the *Rosa26* “safe-harbor” gene (targeted by *sgRosa26*); and the proprotein convertase subtilisin/kexin type 9 (*Pcsk9*) gene (targeted by *sgPcsk9*), a common therapeutic target for lowering circulating cholesterol and reducing the risk of cardiovascular disease (Fig. 1b). Genome-wide off-target predictions for these guides were determined computationally using the Bioconductor package CRISPRseek 1.9.1 [40] with N_4_GN_3_ PAMs and up to six mismatches. Many N_4_GN_3_ PAMS are inactive, so these search parameters are nearly certain to cast a wider net than the true off-target profile. Despite the expansive nature of the search, our analyses revealed no off-target sites with fewer than four mismatches in the mouse genome (Additional file 1: Figure S1). On-target editing efficiencies at these target sites were evaluated in mouse Hepa1–6 hepatoma cells by plasmid transfections and indel quantification was performed by sequence trace decomposition using the Tracking of Indels by Decomposition (TIDE) web tool [41]. We found > 25% indel values for the selected guides, the majority of which were deletions (Fig. 1c).

To evaluate the preliminary efficacy of the constructed all-in-one AAV-sgRNA-hNmeCas9 vector, endotoxin-free *sgPcsk9* plasmid was hydrodynamically administered into the C57Bl/6 mice via tail-vein injection. This method can deliver plasmid DNA to ~ 40% of hepatocytes for transient expression [42]. Indel analyses by TIDE using DNA extracted from liver tissues revealed 5–9% indels 10 days after vector administration (Fig. 1d), comparable to the editing efficiencies obtained with analogous tests of SpyCas9 [43]. These results suggested that NmeCas9 is capable of editing liver cells in vivo.

### Knockout of 4-Hydroxyphenylpyruvate dioxygenase rescues the lethal phenotypes of hereditary Tyrosinemia type I mice

Hereditary Tyrosinemia type I (HT-I) is a fatal genetic disease caused by autosomal recessive mutations in the *Fah* gene, which codes for the fumarylacetoacetate hydroxylase (FAH) enzyme. Patients with diminished FAH have a disrupted tyrosine catabolic pathway, leading to the accumulation of toxic fumarylacetoacetate and succinyl acetoacetate, causing liver and kidney damage [44]. Over the past two decades, the disease has been controlled by 2-(2-Nitro-4-trifluoromethylbenzoyl) -1,3- cyclohexanedione (NTBC), which inhibits 4-hydroxyphenylpyruvate dioxygenase upstream in the tyrosine degradation pathway, thus preventing the accumulation of the toxic metabolites [45]. However, this treatment requires lifelong management of diet and medication and may eventually require liver transplantation [46].

**Fig. 2:**
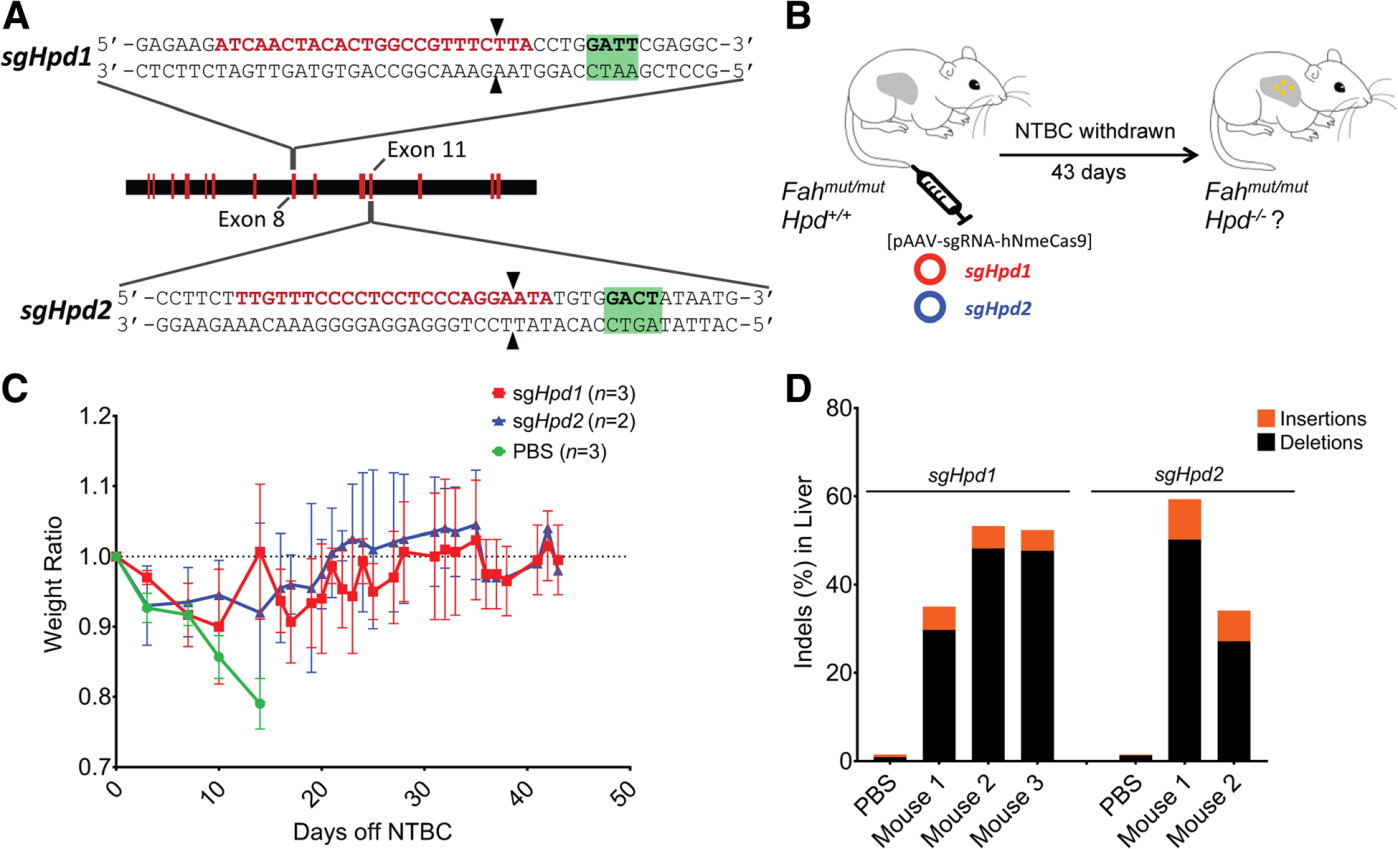
NmeCas9-mediated knockout of *Hpd* rescues the lethal phenotype in Hereditary Tyrosinemia Type I Mice. **a** *Schematic diagram* of the *Hpd* mouse gene. *Red bars* represent exons. Zoomed-in views show the protospacer sequences (*red*) for targeting exon 8 (*sgHpd1*) and exon 11 (*sgHpd2*). NmeCas9 PAM sequences are in *green* and double-stranded break locations are indicated (*black arrowheads*). **b** Experimental design. Three groups of Hereditary Tyrosinemia Type I *Fah*^*−/−*^ mice are injected with PBS or all-in-one AAV-sgRNA-hNmeCas9 plasmids *sgHpd1* or *sgHpd2*. **c** Weight of mice hydrodynamically injected with PBS (*green*), AAV-sgRNA-hNmeCas9 plasmid *sgHpd1* targeting *Hpd* exon 8 (*red*) or *sgHpd2*-targeting *Hpd* exon 11 (*blue*) were monitored after NTBC withdrawal. *Error bars* represent three mice for PBS and *sgHpd1* groups and two mice for the *sgHpd2* group. Data are presented as mean ± SD. **d** *Stacked histogram* showing the percentage distribution of indels at *Hpd* in liver of *Fah*^*−/−*^ mice obtained by TIDE after hydrodynamic injection of PBS or *sgHpd1* and *sgHpd2* plasmids. Livers were harvested at the end of NTBC withdrawal (day 43)

Several gene therapy strategies have been tested to correct the defective *Fah* gene using site-directed mutagenesis [47] or homology-directed repair by CRISPR-Cas9 [47–49]. It has been reported that successful modification of only 1/10,000 of hepatocytes in the liver is sufficient to rescue the phenotypes of *Fah*^*mut/mut*^ mice. Recently, a metabolic pathway reprogramming approach has been suggested in which the function of the hydroxyphenylpyruvate dioxygenase (HPD) enzyme was disrupted by the deletion of exons 3 and 4 of the *Hpd* gene in the liver [35]. This provides us with a context in which to test the efficacy of NmeCas9 editing, by targeting *Hpd* and assessing rescue of the disease phenotype in *Fah* mutant mice [50]. For this purpose, we screened and identified two target sites (one each in exon 8 [*sgHpd1*] and exon 11 [*sgHpd2*]) within the open reading frame of *Hpd* (Fig. 2a). These guides induced average indel efficiencies of 10.8% and 9.1%, respectively, by plasmid transfections in Hepa1–6 cells (Additional file 1: Figure S2).

Three groups of mice were treated by hydrodynamic injection with either phosphate-buffered saline (PBS) or with one of the two *sgHpd1* and *sgHpd2* all-in-one AAV-sgRNA-hNmeCas9 plasmids. One mouse in the *sgHpd1* group and two in the *sgHpd2* group were excluded from the follow-up study due to failed tail-vein injections. Mice were taken off NTBC-containing water seven days after injections and their weight was monitored for 43 days post injection (Fig. 2b). Mice injected with PBS suffered severe weight loss (a hallmark of HT-I) and were sacrificed after losing 20% of their body weight (Fig. 2c). Overall, all *sgHpd1* and *sgHpd2* mice successfully maintained their body weight for 43 days overall and for at least 21 days without NTBC (Fig. 2c). NTBC treatment had to be resumed for 2–3 days for two mice that received *sgHpd1* and one that received *sgHpd2* to allow them to regain body weight during the third week after plasmid injection, perhaps due to low initial editing efficiencies, liver injury due to hydrodynamic injection, or both. Conversely, all other *sgHpd1* and *sgHpd2* treated mice achieved indels with frequencies in the range of 35–60% (Fig. 2d). This level of gene inactivation likely reflects not only the initial editing events but also the competitive expansion of edited cell lineages (after NTBC withdrawal) at the expense of their unedited counterparts [46, 47, 49]. Liver histology revealed that liver damage is substantially less severe in the *sgHpd1*- and *sgHpd2*-treated mice compared to *Fah*^*mut/mut*^ mice injected with PBS, as indicated by the smaller numbers of multinucleated hepatocytes compared to PBS-injected mice (Additional file 1: Figure S3).

### In vivo genome editing by NmeCas9 delivered by a rAAV vector

**Fig. 3:**
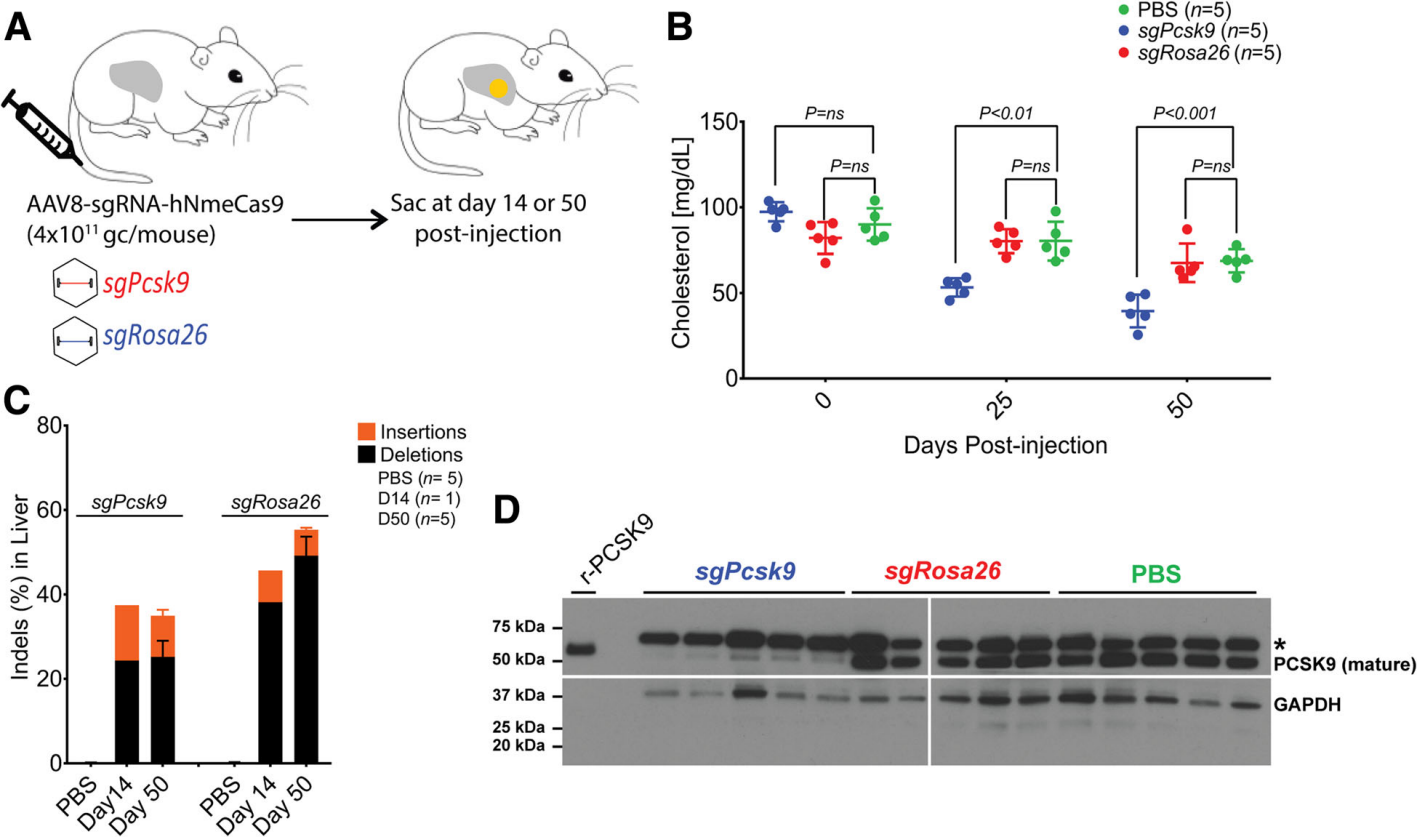
AAV-delivery of NmeCas9 for in vivo genome editing. **a** Experimental outline of AAV8-sgRNA-hNmeCas9 vector tail-vein injections to target *Pcsk9* (*sgPcsk9*) and *Rosa26* (*sgRosa26*) in C57Bl/6 mice. Mice were sacrificed at 14 (*n =* 1) or 50 days (*n = 5*) post injection and liver tissues were harvested. Blood sera were collected at days 0, 25, and 50 post injection for cholesterol level measurement. **b** Serum cholesterol levels. *p* values are calculated by unpaired t test. **c** *Stacked histogram* showing the percentage distribution of indels at *Pcsk9* or *Rosa26* in livers of mice, as measured by targeted deep-sequencing analyses. Data are presented as mean ± SD from five mice per cohort. **d** A representative anti-PCSK9 western blot using total protein collected from day 50 mouse liver homogenates. A total of 2 ng of recombinant mouse PCSK9 (r-PCSK9) was included as a mobility standard. The *asterisk* indicates a cross-reacting protein that is larger than the control recombinant protein

Although plasmid hydrodynamic injections can generate indels, therapeutic development will require less invasive delivery strategies, such as rAAV. To this end, all-in-one AAV-sgRNA-hNmeCas9 plasmids were packaged in hepatocyte-tropic AAV8 capsids to target *Pcsk9* (*sgPcsk9*) and *Rosa26* (*sgRosa26*) (Fig. 1b) [51, 52]. *Pcsk9* and *Rosa26* were used in part to enable NmeCas9 AAV delivery to be benchmarked with that of other Cas9 orthologs delivered similarly and targeted to the same loci [18]. Vectors were administered into C57BL/6 mice via tail vein (Fig. 3a). We monitored cholesterol level in the serum and measured PCSK9 protein and indel frequencies in the liver tissues 25 and 50 days post injection.

Using a colorimetric endpoint assay, we determined that the circulating serum cholesterol level in the *sgPcsk9* mice decreased significantly (*p* < 0.001) compared to the PBS and *sgRosa26* mice at 25 and 50 days post injection (Fig. 3b). Targeted deep-sequencing analyses at *Pcsk9* and *Rosa26* target sites revealed very efficient indels of 35% and 55%, respectively, at 50 days post vector administration (Fig. 3c). Additionally, one mouse of each group was euthanized at 14 days post injection and revealed on-target indel efficiencies of 37% and 46% at *Pcsk9* and *Rosa26*, respectively (Fig. 3c). As expected, PCSK9 protein levels in the livers of *sgPcsk9* mice were substantially reduced compared to the mice injected with PBS and *sgRosa26* (Fig. 3d). The efficient editing, PCSK9 reduction, and diminished serum cholesterol indicate the successful delivery and activity of NmeCas9 at the *Pcsk9* locus.

SpyCas9 delivered by viral vectors is known to elicit host immune responses [19, 53]. To investigate if the mice injected with AAV8-sgRNA-hNmeCas9 generate anti-NmeCas9 antibodies, we used sera from the treated animals to perform IgG1 ELISA. Our results show that NmeCas9 elicits a humoral response in these animals (Additional file 1: Figure S4). Despite the presence of an immune response, NmeCas9 delivered by rAAV is highly functional in vivo, with no apparent signs of abnormalities or liver damage (Additional file 1: Figure S5).

### NmeCas9 is highly specific in vivo

**Fig. 4:**
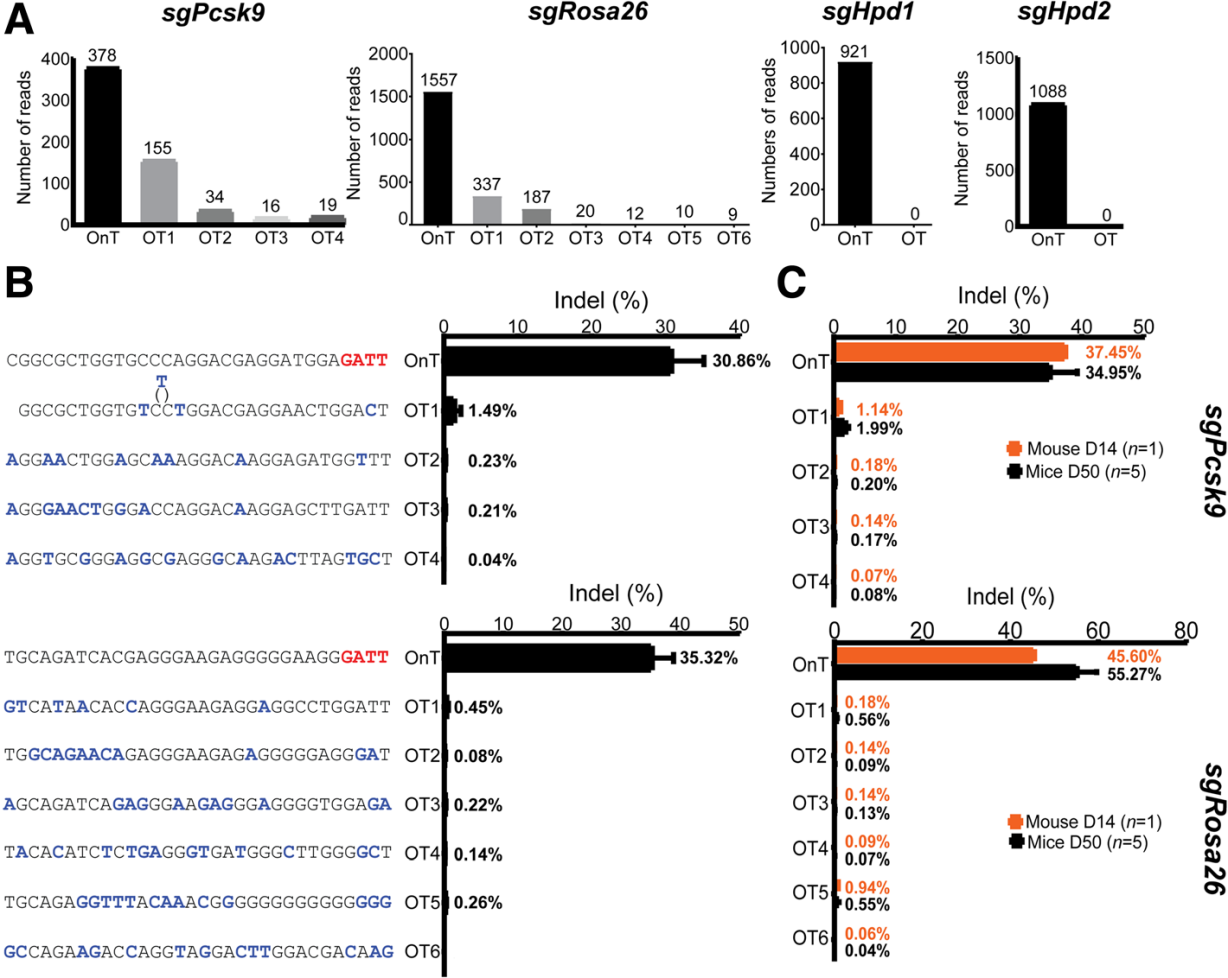
GUIDE-seq genome-wide specificities of NmeCas9. **a** Number of GUIDE-seq reads for the on-target (OnT) and off-target (OT) sites. **b** Targeted deep sequencing to measure the lesion rates at each of the OT sites in Hepa1–6 cells. The mismatches of each OT site with the OnT protospacers is highlighted (*blue*). Data are presented as mean ± SD from three biological replicates. **c** Targeted deep sequencing to measure the lesion rates at each of the OT sites using genomic DNA obtained from mice injected with all-in-one AAV8-sgRNA-hNmeCas9 *sgPcsk9* and *sgRosa26* and sacrificed at day 14 (D14) or day 50 (D50) post injection. Data are presented as mean ± SD

A significant concern in therapeutic CRISPR/Cas9 genome editing is the possibility of off-target edits. We and others have found that wild-type NmeCas9 is a naturally high-accuracy genome editing platform in cultured mammalian cells [32] (Amrani et al., manuscript in revision). To determine if NmeCas9 maintains its minimal off-targeting profile in mouse cells and in vivo, we screened for off-target sites in the mouse genome using genome-wide, unbiased identification of DSBs enabled by sequencing (GUIDE-seq) [22]. Hepa1–6 cells were transfected with *sgPcsk9*, *sgRosa26*, *sgHpd1*, and *sgHpd2* all-in-one AAV-sgRNA-hNmeCas9 plasmids and the resulting genomic DNA was subjected to GUIDE-seq analysis. Consistent with our previous observations in human cells (Amrani et al., manuscript in revision), GUIDE-seq revealed very few off-target (OT) sites in the mouse genome. Four potential OT sites were identified for *sgPcsk9* and another six for *sgRosa26*. We were unable to detect off-target edits with *sgHpd1* and *sgHpd2* (Fig. 4a), thus reinforcing our previous observation that NmeCas9 is often intrinsically hyper-accurate (Amrani et al., manuscript in revision).

Several of the putative OT sites for *sgPcsk9* and *sgRosa26* lack the NmeCas9 PAM preferences (N_4_GATT, N_4_GCTT, N_4_GTTT, N_4_GACT, N_4_GATA, N_4_GTCT, and N_4_GACA) (Fig. 4b) and may therefore represent background. To validate these OT sites, we performed targeted deep sequencing using genomic DNA from Hepa1–6 cells. By this more sensitive readout, indels were undetectable above background at all these OT sites except OT1 of *Pcsk9*, which had an indel frequency < 2% (Fig. 4b). To validate NmeCas9’s high fidelity in vivo, we measured indel formation at these OT sites in liver genomic DNA from the AAV8-NmeCas9-treated, *sgPcsk9-*targeted, and *sgRosa26-*targeted mice. We found little or no detectable off-target editing in mice liver sacrificed at 14 days at all sites except *sgPcsk9* OT1, which exhibited < 2% lesion efficiency (Fig. 4c). More importantly, this level of OT editing stayed below < 2% even after 50 days and also remained either undetectable or very low for all other candidate OT sites. These results suggested that extended (50 days) expression of NmeCas9 in vivo does not compromise its targeting fidelity (Fig. 4c).

## Discussion

### All-in-one rAAV delivery of hNmeCas9

Compared to transcription activator-like effector nucleases (TALENs) and Zinc-finger nucleases (ZFNs), Cas9s are distinguished by their flexibility and versatility [1]. Such characteristics make them ideal for driving the field of genome engineering forward. Over the past few years, CRISPR-Cas9 has been used to enhance products in agriculture, food, and industry, in addition to the promising applications in gene therapy and personalized medicine [54]. Despite the diversity of Class 2 CRISPR systems that have been described, only a handful of them have been developed and validated for genome editing in vivo. In this study, we have shown that NmeCas9 is a compact, high-fidelity Cas9 that can be considered for future in vivo genome editing applications using all-in-one rAAV. Its unique PAM enables editing at additional targets that are inaccessible to the other two compact, all-in-one rAAV-validated orthologs (SauCas9 and CjeCas9).

### Therapeutic gene correction for hereditary Tyrosinemia type 1 by metabolic pathway reprograming

Patients with mutations in the *HPD* gene are considered to have Type III Tyrosinemia and exhibit high level of tyrosine in blood, but otherwise appear to be largely asymptomatic [55, 56]. HPD acts upstream of FAH in the tyrosine catabolism pathway and *Hpd* disruption ameliorates HT-I symptoms by preventing the toxic metabolite build-up that results from loss of FAH. Structural analyses of HPD reveal that the catalytic domain of the HPD enzyme is located at the C-terminus of the enzyme and encoded by exon 13 and 14 [57]. Thus, frameshift-inducing indels upstream of exon 13 should render the enzyme inactive. We used this context to demonstrate that *Hpd* inactivation by hydrodynamic injection of NmeCas9 plasmid is a viable approach to rescue HT-I mice. NmeCas9 can edit sites carrying several different PAMs (N_4_GATT [consensus], N_4_GCTT, N_4_GTTT, N_4_GACT, N_4_GATA, N_4_GTCT, and N_4_GACA) (Amrani et al., manuscript in revision). Our *Hpd* editing experiments confirmed one of the variant PAMs in vivo with the *sgHpd2* guide, which targets a site with a N_4_GACT PAM.

### Efficient, accurate NmeCas9 genome editing with rAAV delivery

To achieve targeted delivery of NmeCas9 to various tissues in vivo, rAAV vectors are a promising delivery platform due to the compact size of NmeCas9 transgene, which allows the delivery of NmeCas9 and its guide in an all-in-one format. We have validated this approach for the targeting of *Pcsk9* and *Rosa26* genes in adult mice, with efficient editing observed even at 14 days post injection. As observed previously in cultured cells [32] (Amrani et al., manuscript in revision), NmeCas9 is intrinsically accurate, even without the extensive engineering that was required to reduce off-targeting by SpyCas9 [21–23]. We performed side-by-side comparisons of NmeCas9 OT editing in cultured cells and in vivo by targeted deep sequencing and we found that off-targeting is minimal in both settings. Editing at the *sgPcsk9* OT1 site (within an unannotated locus) was the highest detectable at ~ 2%. Despite these promising results, more extensive and long-term studies, including in larger animals, will be needed to fully understand the long-term effects of Cas9 expression in tissues, as well as the development of approaches that clear viral vectors after editing is complete.

## Conclusions

We demonstrate that NmeCas9 is amenable to in vivo genome editing using the highly desirable all-in-one rAAV platform. With its unique PAM preferences and high fidelity, this all-in-one AAV-sgRNA-hNeCas9 can be applied to a range of genome editing purposes in vivo. We anticipate that successful in vivo delivery of this accurate and effective Cas9 can advance therapeutic editing in humans.

## Methods

### Construction of all-in-one AAV-sgRNA-hNMeCas9 plasmid and rAAV vector production

The human-codon-optimized NmeCas9 gene under the control of the U1a promoter and a sgRNA cassette driven by the U6 promoter were cloned into an AAV2 plasmid backbone. The NmeCas9 ORF was flanked by four nuclear localization signals – two on each terminus – in addition to a triple-HA epitope tag. This plasmid is available through Addgene (plasmid ID 112139). Oligonucleotides with spacer sequences targeting *Hpd*, *Pcsk9*, *and Rosa26* were inserted into the sgRNA cassette by ligation into a SapI cloning site (Additional file 2).

AAV vector production was performed at the Horae Gene Therapy Center at the University of Massachusetts Medical School. Briefly, plasmids were packaged in AAV8 capsid by triple-plasmid transfection in HEK293 cells and purified by sedimentation as previously described [58].

The off-target profiles of these spacers were predicted computationally using the Bioconductor package CRISPRseek. Search parameters were adapted to NmeCas9 settings as described previous (Amrani et al., manuscript in revision): gRNA.size = 24, PAM = “NNNNGATT,” PAM.size = 8, RNA.PAM.pattern = “NNNNGNNN$,” weights = c(0, 0, 0, 0, 0, 0, 0.014, 0, 0, 0.395, 0.317, 0, 0.389, 0.079, 0.445, 0.508, 0.613, 0.851, 0.732, 0.828, 0.615, 0.804, 0.685, 0.583), max.mismatch = 6, allowed.mismatch.PAM = 7, topN = 10,000, min.score = 0.

### Cell culture and transfection

Mouse Hepa1–6 hepatoma cells were cultured in DMEM with 10% fetal bovine serum and 1% Penicillin/Streptomycin (Gibco) in a 37 °C incubator with 5% CO_2_. Transient transfections of Hepa1–6 cells were performed using Lipofectamine LTX. For transient transfection, approximately 1 × 10^5^ cells per well were cultured in 24-well plates 24 h before transfection. Each well was transfected with 500-ng all-in-one AAV-sgRNA-hNmeCas9 plasmid, using Lipofectamine LTX with Plus Reagent (Invitrogen) according to the manufacturer’s protocol.

### DNA isolation from cells and liver tissue

Genomic DNA isolation from cells was performed 72 h post transfection using DNeasy Blood and Tissue kit (Qiagen) following the manufacturer’s protocol. Mice were sacrificed and liver tissues were collected 10 days post hydrodynamic injection or 14 and 50 days post tail-vein rAAV injection. Genomic DNA was isolated using DNeasy Blood and Tissue kit (Qiagen) according to the manufacturer’s protocol.

### GUIDE-seq

GUIDE-seq analysis was performed as previously described [22]. Briefly, 7.5 pmol of annealed GUIDE-seq oligonucleotides and 500 ng of all-in-one AAV-sgRNA-hNmeCas9 plasmids targeting *Pcsk9*, *Rosa26*, and *Hpd* were transfected into 1 × 10^5^ Hepa1–6 cells using Lipofectamine LTX with Plus Reagent (Invitrogen). At 72 h post transfection, genomic DNA was extracted using a DNeasy Blood and Tissue kit (Qiagen) per manufacturer protocol. Library preparations, deep sequencing, and reads analysis were performed as previously described [59, 60]. The Bioconductor package GUIDEseq was used for off-target analysis as described previously using maximum allowed mismatch of 10 nt between the guide and target DNA [59]. For read alignment, mouse mm10 was used as a reference genome.

### Indel analysis

TIDE primers were designed ~ 700 bp apart, with the forward primer at ~ 200 bp upstream of the cleavage site. A total of 50 ng of genomic DNA was used for PCR amplification with High Fidelity 2X PCR Master Mix (New England Biolabs). For TIDE analysis, 30 μL of a PCR product was purified using QIAquick PCR Purification Kit (Qiagen) and sent for Sanger sequencing using the TIDE forward primer (Additional file 3). Indel values were obtained using the TIDE web tool (https://tide-calculator.nki.nl/) as described previously [41].

Targeted deep-sequencing analysis was performed for Hepa1–6 cells and mouse liver gDNA using a two-step PCR amplification approach as described previously [60] (Amrani et al., manuscript in revision). Briefly, in the first PCR step, on-target or off-target locus-specific primers were used to amplify the editing site using Phusion High Fidelity DNA Polymerase (New England Biolabs) with a 65 °C annealing temperature. The primer ends contained sequences complementary to Illumina TruSeq adaptor sequences (Additional file 3). In the second-step PCR, equimolar amounts of DNA were amplified with a universal forward primer and an indexed reverse primer using Phusion High Fidelity DNA Polymerase (98 °C, 15 s; 61 °C, 25 s; 72 °C, 18 s; nine cycles) to ligate the TruSeq adaptors. The resultant amplicons were separated in a 2.5% agarose gel and the corresponding ~ 250-bp product bands were extracted using Monarch DNA Gel Extraction Kit (New England Biolabs).

The libraries were then sequenced on an Illumina MiSeq in paired-end mode with a read length of 150 bp. To analyze genome editing outcomes at genomic sites, the command line utilities of CRISPResso were used [61]. Input parameters were adjusted to filter low-quality reads (−q 30 -s 20). Furthermore, the background was determined using the control sample (no guide) and subtracted from the experimental samples. The resulting indel frequencies, sizes, and distributions were then plotted using Graphpad PRISM.

### Animals and liver tissue processing

For hydrodynamic injections, 2.5 mL of 30 μg of endotoxin-free AAV-sgRNA-hNmeCas9 plasmid targeting *Pcsk9* or 2.5 mL PBS was injected by tail vein into 9- to 18-week-old female C57BL/6 mice. Mice were euthanized 10 days later and liver tissue was harvested. For the AAV8 vector injections, 12- to 16-week-old female C57BL/6 mice were injected with 4 × 10^11^ genome copies per mouse via tail vein, using vectors targeting *Pcsk9* or *Rosa26*. Mice were sacrificed 14 and 50 days after vector administration and liver tissues were collected for analysis.

For *Hpd* targeting, 2 mL PBS or 2 mL of 30 μg of endotoxin-free AAV-sgRNA-hNmeCas9 plasmid was administered into 15- to 21-week-old Type 1 Tyrosinemia *Fah* knockout mice (*Fah*^*neo*^) via tail vein. The encoded sgRNAs targeted sites in exon 8 (*sgHpd1*) or exon 11 (*sgHpd2*). The HT1 homozygous mice with the *Fah*^*neo*^ allele in a 129 background were kindly provided by Dr. Markus Grompe [50]. The HT1 mice were fed with 10 mg/L NTBC (2-(2-nitro-4-trifluoromethylbenzoyl)-1,3-cyclohexanedione) (Sigma-Aldrich, Cat. No. PHR1731-1G) in drinking water when indicated. Both sexes were used in these experiments. Mice were maintained on NTBC water for seven days post injection and then switched to normal water. Body weight was monitored every 1–3 days. The PBS-injected control mice were sacrificed when they became moribund after losing 20% of their body weight after removal from NTBC treatment.

Mice were euthanized according to our protocol and liver tissue was sliced and fragments stored at − 80 °C. Some liver tissues were fixed in 4% formalin overnight, embedded in paraffin, sectioned and stained with hematoxylin and eosin (H&E).

### Serum analysis

Blood (~ 200 μL) was drawn from the facial vein at 0, 25, and 50 days post vector administration. Serum was isolated using a serum separator (BD, Cat. No. 365967) and stored under − 80 °C until assay.

Serum cholesterol levels were measured by Infinity™ colorimetric endpoint assay (Thermo-Scientific) following the manufacturer’s protocol. Briefly, serial dilutions of Data-Cal™ Chemistry Calibrator were prepared in PBS. In a 96-well plate, 2 μL of mice sera or calibrator dilution was mixed with 200 μL of Infinity™ cholesterol liquid reagent, then incubated at 37 °C for 5 min. The absorbance was measured at 500 nm using a BioTek Synergy HT microplate reader.

### Western blot

Liver tissue fractions were ground and resuspended in 150 μL of RIPA lysis buffer. Total protein content was estimated by Pierce™ BCA Protein Assay Kit (Thermo-Scientific) following the manufacturer’s protocol. A total of 20 μg of protein from tissue or 2 ng of Recombinant Mouse Proprotein Convertase 9/PCSK9 Protein (R&D Systems, 9258-SE-020) were loaded onto a 4–20% Mini-PROTEAN® TGX™ Precast Gel (Bio-Rad). The separated bands were transferred onto PVDF membrane and blocked with 5% Blocking-Grade Blocker solution (Bio-Rad) for 2 h at room temperature. Membranes were incubated with rabbit anti-GAPDH (Abcam ab9485, 1:2000) or goat anti-PCSK9 (R&D Systems AF3985, 1:400) antibodies overnight at 4 °C. Membranes were washed five times in TBST and incubated with horseradish peroxidase (HRP)-conjugated goat anti-rabbit (Bio-Rad 1,706,515, 1:4000) and donkey anti-goat (R&D Systems HAF109, 1:2000) secondary antibodies for 2 h at room temperature. The membranes were washed five times in TBST and visualized with Clarity™ western ECL substrate (Bio-Rad) using an M35A X-OMAT Processor (Kodak).

### Humoral immune response

Humoral IgG1 immune response to NmeCas9 was measured by ELISA (Bethyl; Mouse IgG1 ELISA Kit, E99–105) following manufacturer’s protocol with a few modifications. Briefly, expression and three-step purification of NmeCas9 and SpyCas9 was performed as previously described [4]. A total of 0.5 μg of recombinant NmeCas9 or SpyCas9 proteins suspended in 1× coating buffer (Bethyl) were used to coat 96-well plates (Corning) and incubated for 12 h at 4 °C with shaking. The wells were washed three times while shaking for 5 min using 1× Wash Buffer. Plates were blocked with 1× BSA Blocking Solution (Bethyl) for 2 h at room temperature, then washed three times. Serum samples were diluted 1:40 using PBS and added to each well in duplicate. After incubating the samples at 4 °C for 5 h, the plates were washed 3× times for 5 min and 100 μL of biotinylated anti-mouse IgG1 antibody (Bethyl; 1: 100,000 in 1 x BSA Blocking Solution) was added to each well. After incubating for 1 h at room temperature, the plates were washed four times and 100 μL of TMB Substrate was added to each well. The plates were allowed to develop in the dark for 20 min at room temperature and 100 μL of ELISA Stop Solution was then added per well. Following the development of the yellow solution, absorbance was recorded at 450 nm using a BioTek Synergy HT microplate reader.

## Additional files

Additional file 1: **Figure S1.** Genome-wide computational prediction of NmeCas9 off-target sites using CRISPRseek with the N_4_GN_3_ PAM. Search parameters were set to identify sites with up to six mismatches to the spacer sequence. The total number of detected off-target sites for each protospacer is indicated. **Figure S2.** Stacked histogram showing the percentage distribution of indels obtained by TIDE after AAV-sgRNA-hNmeCas9 plasmids transfections in Hepa1–6. Error bars represent three independent experiments. Data are presented as mean ± SD. **Figure S3.** H&E staining from wild-type (*Fah*^*+/+*^) mouse, and HT-I mice (*Fah*^*−/−*^) injected with PBS or AAV-sgRNA-hNmeCas9 plasmids *sgHpd1* or *sgHpd2*. Scale bar is 20 μm. **Figure S4.** Humoral IgG1 immune response to NmeCas9 in vivo. Serum collected at day 50 post injection with all-in-one AAV8-sgRNA-hNmeCas9 *sgPcsk9* and *sgRosa26*. Serum antibodies reacted against NmeCas9 protein (left) or SpyCas9 protein (right). **Figure S5.** H&E staining from PBS, *sgPcsk9*, and *sgRosa26* AAV8 injected mice. Scale bar is 20 μm. (PDF 713 kb)

Additional file 2: Nucleotide sequence of all-in-one AAV-sgRNA-hNmeCas9 Plasmid. (PDF 59 kb)

Additional file 3: Contains Protospacer sequences, TIDE Primers sequences, and Deep-sequencing Primers sequences. (PDF 69 kb)

## Abbreviations

AAV: Adeno-associated virus
bp: Base pair
Cas: CRISPR-associated
CjeCas9: *Campylobacter jejuni* Cas9
CRISPR: Clustered, regularly interspaced, short palindromic repeats
crRNAs: CRISPR RNAs
dCas9: “Dead” Cas9
DSB: Double-strand breaks
dsODN: Double-stranded oligodeoxynucleotide
FAH: Fumarylacetoacetate hydroxylase
GUIDE-seq: Genome-wide unbiased identification of double strand breaks enabled by sequencing
HDR: Homology-directed repair
HPD: Hydroxyphenylpyruvate dioxygenase
HTI: Hereditary tyrosinemia type I
IRD: Inherited retinal disease
LDL: Low-density lipoprotein
MGEs: Mobile genetic elements
NHEJ: Non-homologous end joining
NLS: Nuclear localization signal
NmeCas9: *Neisseria meningitidis* (strain 8013) Cas9
NTBC: 2-(2-Nitro-4-trifluoromethylbenzoyl) -1,3- cyclohexanedione
NTS: NmeCas9 target site
PAM: Protospacer adjacent motif
PCSK9: Proprotein convertase subtilisin/kexin type 9
rAAV: Recombinant adeno-associated virus
RNP: Ribonucleoprotein
SauCas9: *Staphylococcus aureus* Cas9
sgRNA: Single-guide RNA
SpyCas9: *Streptococcus pyogenes* Cas9
TALENs: Transcription activator-like effector nucleases
TIDE: Tracking of indels by decomposition
tracrRNA: *Trans*-acting CRISPR RNA
ZFNs: Zinc-finger nucleases
