# Supplementary figures and images for: All-in-one adeno-associated virus delivery and genome editing by Neisseria meningitidis Cas9 in vivo
Source: Genome Biol. 2018 Sep 19;19:137. doi: 10.1186/s13059-018-1515-0 (PMC6146650; doi:10.1186/s13059-018-1515-0)

# Additional File 1

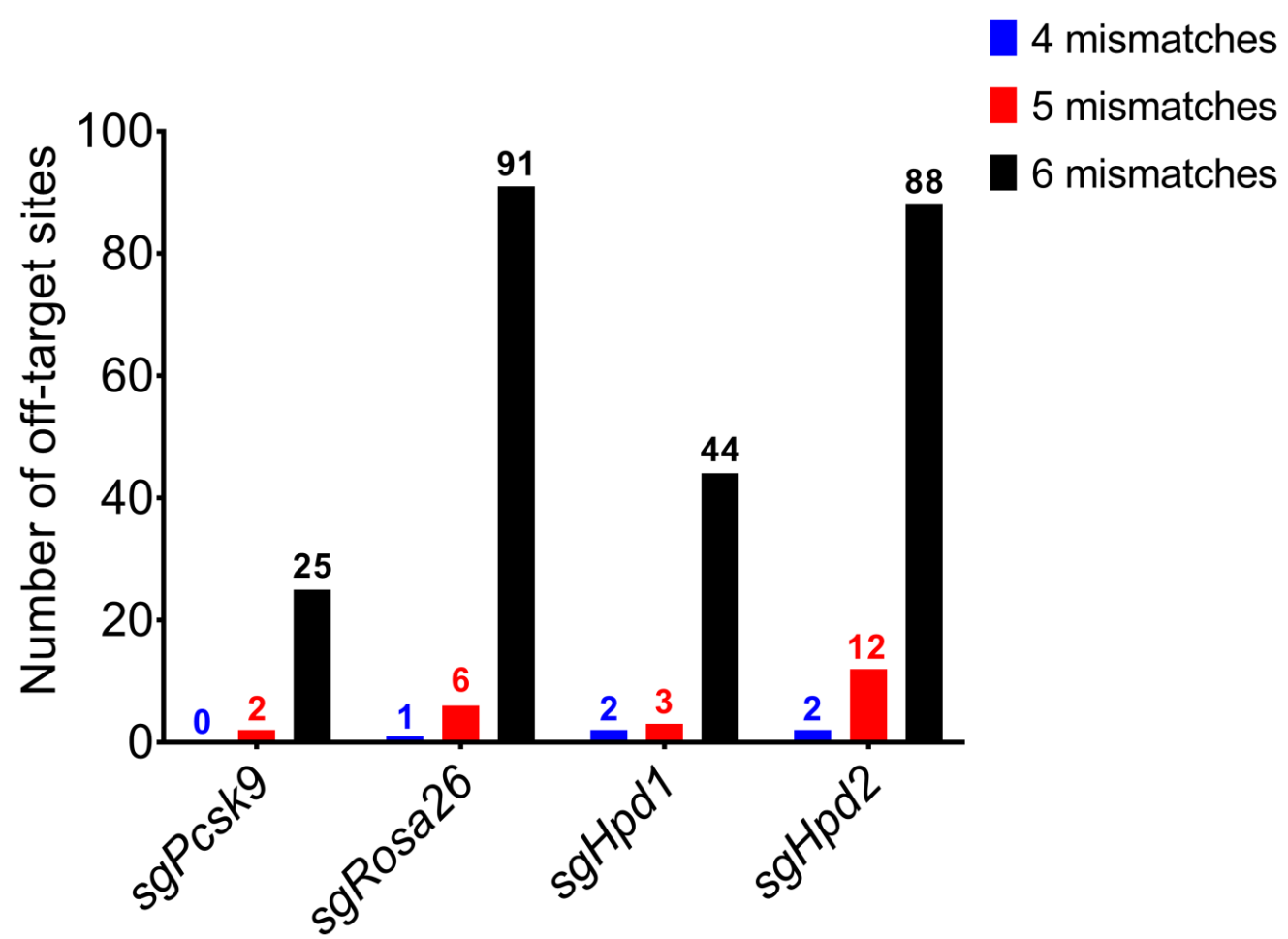

Fig. S1

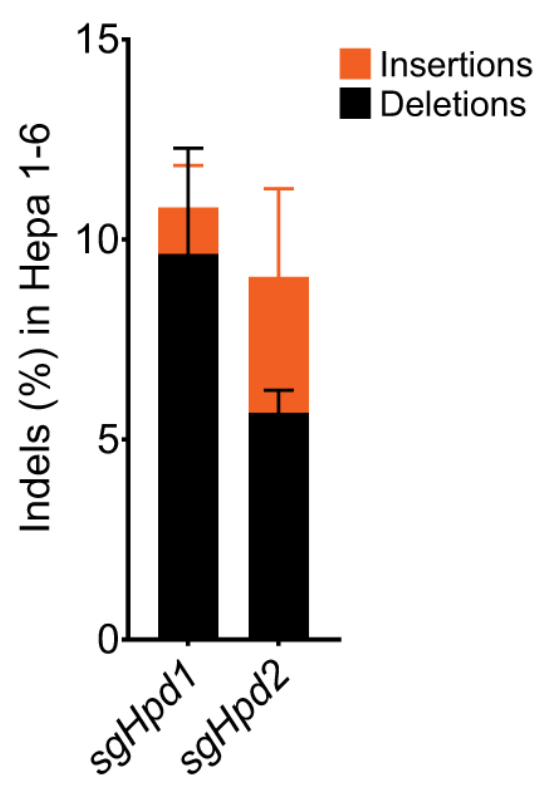

**Fig. S2**

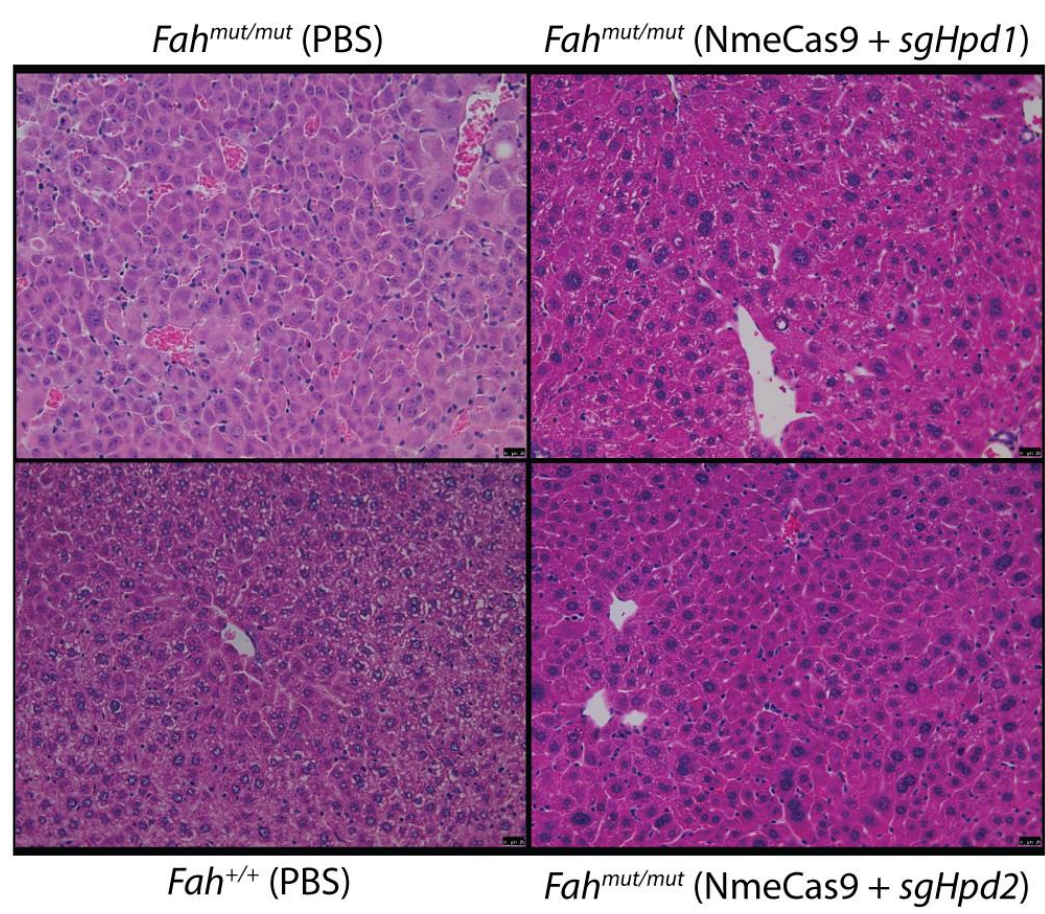

**Fig. S3**

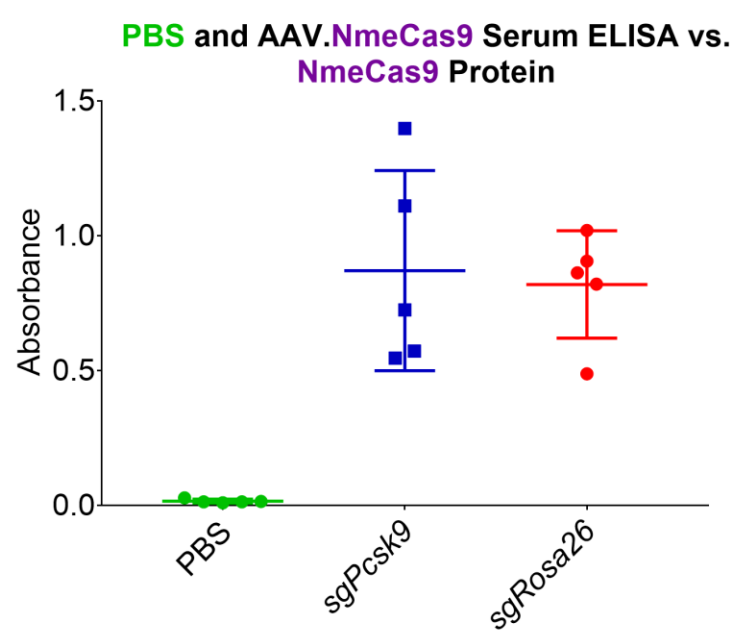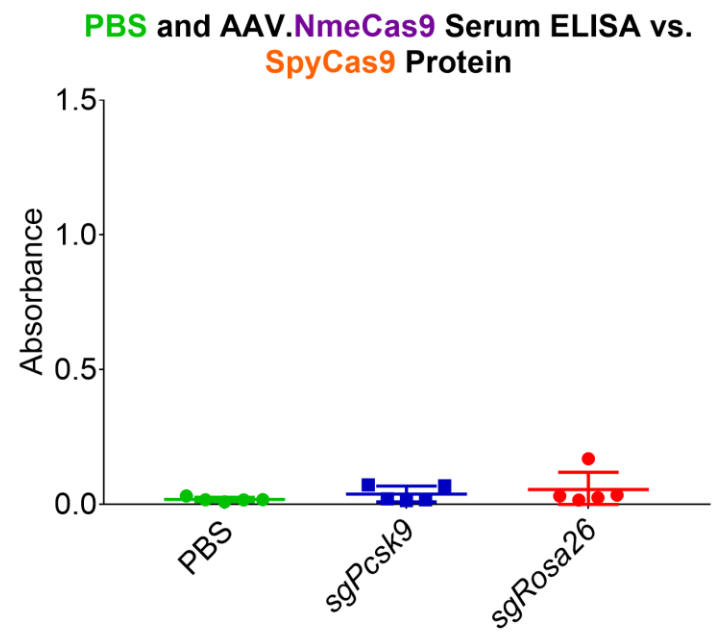

**Fig. S4**

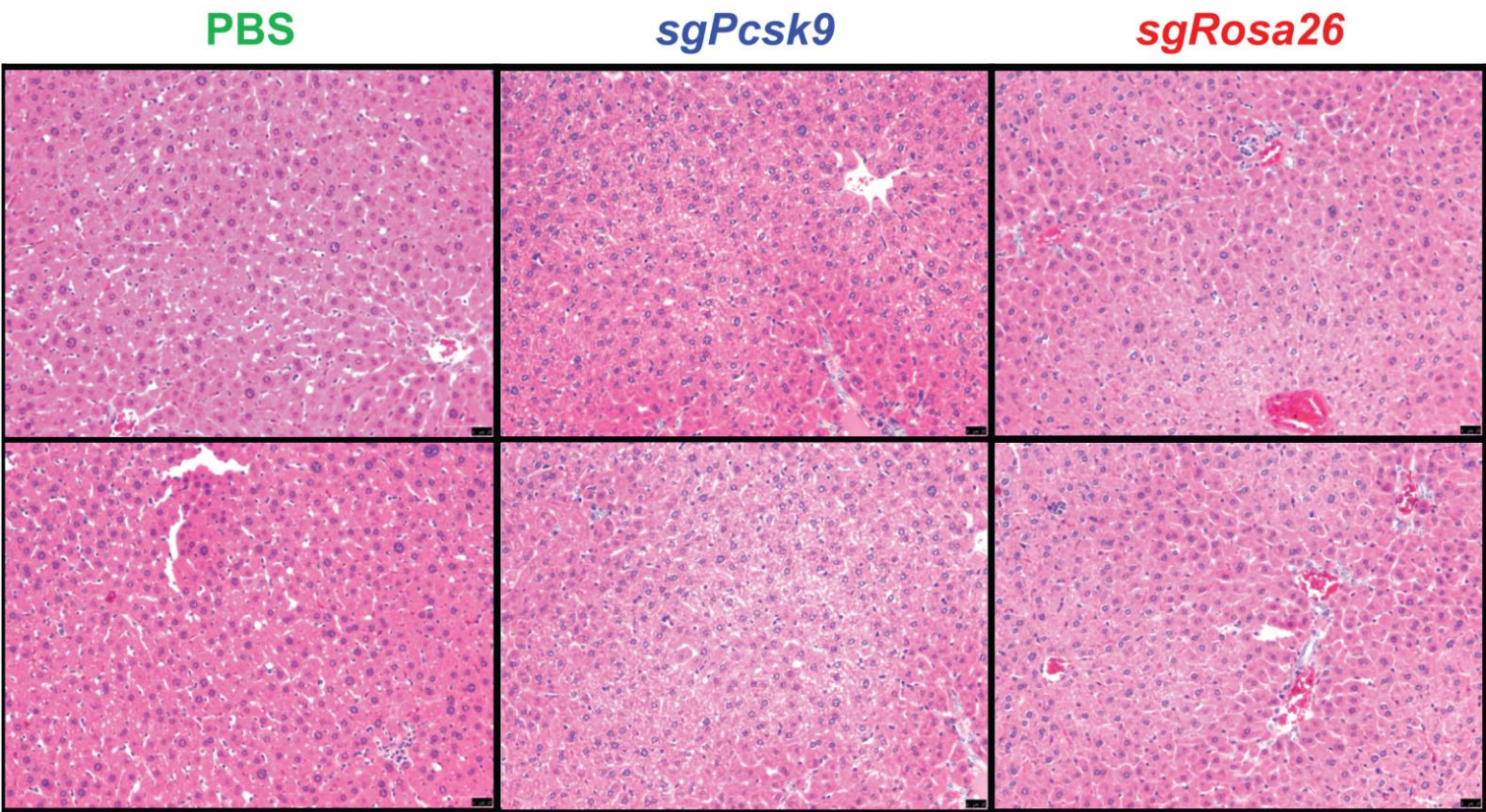

Fig. S5

Supplement: Supplementary file 1 — Figure S1. Genome-wide computational prediction of NmeCas9 off-target sites using CRISPRseek with the N4GN3 PAM. Search parameters were set to identify sites with up to six mismatches to the spacer sequence. The total number of detected off-target sites for each protospacer is indicated. Figure S2. Stacked histogram showing the percentage distribution of indels obtained by TIDE after AAV-sgRNA-hNmeCas9 plasmids transfections in Hepa1–6. Error bars represent three independent experiments. Data are presented as mean ± SD. Figure S3. H&E staining from wild-type (Fah+/+) mouse, and HT-I mice (Fah−/−) injected with PBS or AAV-sgRNA-hNmeCas9 plasmids sgHpd1 or sgHpd2. Scale bar is 20 μm. Figure S4. Humoral IgG1 immune response to NmeCas9 in vivo. Serum collected at day 50 post injection with all-in-one AAV8-sgRNA-hNmeCas9 sgPcsk9 and sgRosa26. Serum antibodies reacted against NmeCas9 protein (left) or SpyCas9 protein (right). Figure S5. H&E staining from PBS, sgPcsk9, and sgRosa26 AAV8 injected mice. Scale bar is 20 μm. (PDF 713 kb) [file 13059_2018_1515_MOESM1_ESM.pdf]
