# Supplementary material for: All-in-one adeno-associated virus delivery and genome editing by Neisseria meningitidis Cas9 in vivo
Source: Genome Biol. 2018 Sep 19;19:137. doi: 10.1186/s13059-018-1515-0 (PMC6146650; doi:10.1186/s13059-018-1515-0)
Supplement: Supplementary file 3 — Contains Protospacer sequences, TIDE Primers sequences, and Deep-sequencing Primers sequences. (PDF 69 kb) [file 13059_2018_1515_MOESM3_ESM.pdf]

Additional file 3:

Protospacer sequences

Name	Gene	Chr	Protospacer Sequence
**sgHpd1**	*Hpd*	chr5:-:123178208:123178239	ATCAACTACACTGGCCGTTTCTTACCTGGATT
**sgHpd2**	*Hpd*	chr5:-:123175950:123175981	TTGTTTCCCCTCCTCCCAGGAATATGTGGACT
**sgPcsk9**	*Pcsk9*	chr4:-:106463790:106463821	CGGCGCTGGTGCCCAGGACGAGGATGGAGATT
**sgRosa26**	*Rosa26*	chr6:+:113076048:113076079	TGCAGATCACGAGGGAAGAGGGGGAAGGGATT
**sgPcsk9_OT1**	*unannotated*	chr6:-:24894402:24894433	GGCGCTGTGTGTCCTGGACGAGGAACTGGACT
**sgPcsk9_OT2**	*Rnf214*	chr9:+:45868164:45868195	AGGAACTGGAGCAAAGGACAAggagatggttt
**sgPcsk9_OT3**	*Pcsk9*	chr4:-:106463561:106463592	AGGTGCGGGAGGCGAGGGCAAGACTTAGTGCT
**sgPcsk9_OT4**	*unannotated*	chr16:+:47959998:47960029	AGGGAACTGGGACCAGGACAAGGAGCTTGATT
**sgRosa26_OT1**	*Ptcd2*	chr13:-:99335395:99335426	gtcataacaccagggaagaggaggcctggatt
**sgRosa26_OT2**	*unannotated*	chr1:-:96248259:96248290	TGGCAGAACagagggaagagagggggagggat
**sgRosa26_OT3**	*Tctn3*	chr19:-:40606453:40606484	AGCAGATCAGAGGGAAGAGGGAGGGGTGGAGA
**sgRosa26_OT4**	*unannotated*	chr3:-:121042039:121042070	TACACATCTCTGAGGGTGATGGGCTTGGGGCT
**sgRosa26_OT5**	*unannotated*	chr10:-:8135358:8135389	TGCAGAGGTTTACAAACggggggggggggggg
**sgRosa26_OT6**	*Sergef*	chr7:-:46544656:46544687	gccagaagaccaggtaggacttggacgacaag

TIDE Primers

Target Site	5’ Primer	3’ Primer
**sgHpd1**	GGCAGGTAGTCGAACATTCC	CAGCCGTGGTTAACTGAACA
**sgHpd2**	CTGTGGTGTGCATGATGTC	CAGCAAAATACCAGCGG
**sgPcsk9**	GGCTCCCGTTCTCTCTCTCT	CGCTAAATCGAGGCCTACAG
**sgRosa26**	TCAGTTGGGCTGTTTTGGAG	TAGGGGTTGGATAAGCCAGT

Deep-sequencing Primers

Target Site	5' Primer	3' Primer
**sgPcsk9**	ctacacgacgctcttccgatctGCTACTGTGCCCCACCGG	agacgtgtgctcttccgatctCTCATCAGCCAGGCCATC
**sgPcsk9_OT1**	ctacacgacgctcttccgatctGTGAGGTGCCAGAGTGGTGT	agacgtgtgctcttccgatctGGCTGTCAGGGTGAGAAGTG
**sgPcsk9_OT2**	ctacacgacgctcttccgatctCGACAAAAGAGATGAGGAGAAAA	agacgtgtgctcttccgatctTGAACTCAAGTCCTTATGCTTGC
**sgPcsk9_OT3**	ctacacgacgctcttccgatctCCTCCCCCATCACTCTGTG	agacgtgtgctcttccgatctTCTAGCCGTCTGGTGGTCTC
**sgPcsk9_OT4**	ctacacgacgctcttccgatctTTTGAAATGCAATCATAGCACA	agacgtgtgctcttccgatctGAGACAGTAGAGCAGAATGAAGGA
**sgRosa26**	ctacacgacgctcttccgatctCTTCTGAGGACCGCCCT	agacgtgtgctcttccgatctGCCTTTAAGCCTGCCCAG
**sgRosa26_OT1**	ctacacgacgctcttccgatctCACTGGCTTGGAACACACAA	agacgtgtgctcttccgatctGCCCACGGTTTCAGAAAGTA
**sgRosa26_OT2**	ctacacgacgctcttccgatctTCTCTGCCATTTCCTCTTCC	agacgtgtgctcttccgatctAAGCCCATGATTTTGTGAAA
**sgRosa26_OT3**	ctacacgacgctcttccgatctATGGAGAAGCCTCCCTTCAC	agacgtgtgctcttccgatctCCCTAAGTTTAACCCTCAGCA
**sgRosa26_OT4**	ctacacgacgctcttccgatctGACAAATATGAGTTTTTACTACCAGGA	agacgtgtgctcttccgatctCGTCTTAATGAGCCCGATCT
**sgRosa26_OT5**	ctacacgacgctcttccgatctTCAGGAAACACTTCCGCTTC	agacgtgtgctcttccgatctCCGAACATCAGGATGTGAAA
**sgRosa26_OT6**	ctacacgacgctcttccgatctGAGCTTTCTGCCTCTGTGCT	agacgtgtgctcttccgatctGGAGGAGGTGCTATCTGTGC
